# Bacterial Agents Detected in 418 Ticks Removed from Humans during 2014–2021, France

**DOI:** 10.3201/eid2904.221572

**Published:** 2023-04

**Authors:** Marie Jumpertz, Jacques Sevestre, Léa Luciani, Linda Houhamdi, Pierre-Edouard Fournier, Philippe Parola

**Affiliations:** Aix-Marseille University, Marseille, France; IHU-Méditerranée Infection, Marseille

**Keywords:** ticks, bacteria, parasites, Rickettsia, vector-borne infections, zoonoses, Borrelia, Anaplasma, MALDI-TOF MS, mass spectrometry, SENLAT, Dermacentor, Rhipicephalus, Ixodes, Amblyomma, Hyalomma, France

## Abstract

Monitoring of tickborne diseases is critical for prevention and management. We analyzed 418 ticks removed from 359 patients during 2014–2021 in Marseille, France, for identification and bacteria detection. Using morphology, molecular methods, or matrix-assisted laser desorption/ionization time-of-flight mass spectrometry, we identified 197 (47%) *Ixodes*, 136 (33%) *Dermacentor*, 67 (16%) *Rhipicephalus*, 8 (2%) *Hyalomma*, 6 (1%) *Amblyomma*, 2 (0.5%) *Argas*, and 2 (0.5%) *Haemaphysalis* tick species. We also detected bacterial DNA in 241 (58%) ticks. The most frequent bacterial pathogens were *Rickettsia* *raoultii* (17%) and *R.* *slovaca* (13%) in *Dermacentor* ticks, *Borrelia* spp. (9%) in *Ixodes* ticks, and *R.* *massiliae* (16%) in *Rhipicephalus* ticks. Among patients who were bitten, 107 had symptoms, and tickborne diseases were diagnosed in 26, including scalp eschar and neck lymphadenopathy after tick bite and Lyme borrelioses. Rapid tick and bacteria identification using a combination of methods can substantially contribute to clinical diagnosis, treatment, and surveillance of tickborne diseases.

Ticks are obligate hematophagous arthropods that are the second most prevalent vectors of human zoonotic pathogens after mosquitoes (*1*). Ticks harbor a vast number of pathogenic bacteria, viruses, and protozoa (*2*). Emerging tickborne disease (TBD) agents that cause human infections, such as *Borrelia* *miyamotoi* and *Rickettsia* *tamurae*, have been reported (*3*). In Europe, ticks most frequently implicated in human infectious diseases are *Ixodes*, *Rhipicephalus*, and *Dermacentor* spp. (*4*). Tularemia, Crimean-Congo hemorrhagic fever, and tick-borne encephalitis are the 3 TBDs under specific surveillance by the European Centre for Disease Prevention and Control (*5*). Surveillance of other TBDs relies mainly on national reference centers, reports, literature analysis, and serologic surveys. Most TBDs have geographic disease patterns, and distribution evolves with climatic conditions and human behavioral modifications. Monitoring TBDs is critical for developing optimal prevention and management strategies (*6*). 

The Institut Hospitalo-Universitaire Méditerranée Infection (IHU-MI) in Marseille, France (*7*) includes the National Reference Centre for rickettsioses and bartonelloses and Southern Reference Center for tickborne diseases. The laboratory receives ticks collected from the field, animals, and patients in France and worldwide. Analyses include species-level tick identification and detection of tickborne human bacterial pathogens. Clinicians or patients are contacted to use this information for surveillance, medical advice, and treatment.

In 2016, a study of tickborne bacteria and ticks removed from humans during 2002–2013 was conducted at the Aix-Marseille University (*8*). Innovative entomologic and microbiologic techniques were used for identification of ticks and bacteria, including molecular methods and matrix-assisted laser desorption/ionization time-of-flight (MALDI-TOF) mass spectrometry. The principle of MALDI-TOF mass spectrometry identification resides in acquisition of species-specific mass spectra from a study sample. Spectra are secondarily queried against a reference mass spectral database, enabling identification on the basis of the spectra’s similarity profile (*9*). This identification technique revolutionized everyday practices in clinical microbiology laboratories and has proven to be a robust, reproducible, and time-effective method for identifying arthropod vectors, notably ticks (*9*). We analyzed 418 ticks removed from humans and sent to the IHU-MI during 2014–2021, using MALDI-TOF mass spectrometry to identify the ticks and molecular methods and serology to identify tickborne pathogenic bacteria.

## Material and Methods

### Tick Identification

We included all ticks removed from humans and analyzed at the IHU-MI during January 2014–March 2021. When possible, the tick was first identified morphologically by an entomologist by using morphologic identification keys applicable to the specimen’s geographic location (*10*,*11*). When available, we used 4 legs of each tick to identify the tick species by using MALDI-TOF mass spectrometry (*9*). We obtained protein mass profiles for each sample by using the Microflex LT MALDI-TOF instrument (Bruker, https://www.bruker.com) as described (*12*). We compared protein spectra to those in our in-house arthropod MALDI-TOF mass spectrometry database. For molecular identification of ticks, we extracted DNA from half of the tick body by using the EZ1 DNA Tissue Kit (QIAGEN, https://www.qiagen.com) (*12*) and performed Sanger sequencing of a 360-bp PCR amplification product from the 12S rRNA gene (*13*).

### Detecting and Identifying Bacteria in Ticks

We identified tickborne bacteria by using the same DNA extracts used for molecular identification of ticks. We used DNA samples extracted from uninfected laboratory-reared *Rhipicephalus* *sanguineus* s.l. ticks as negative controls. We screened ticks for *Rickettsia* spp., *Bartonella* spp., *Borrelia* spp., *Francisella* *tularensis*, *Coxiella* *burnetii*, *Coxiella*-like bacteria, and Anaplasmataceae bacteria by using quantitative real-time or standard PCR (Table 1; Appendix Table).

**Table 1:** Tick identification and number of bacterial agents detected in 418 ticks removed from humans during 2014–2021 in France and their geographic origin*

Tick species	No. ticks	Tick origin	Bacteria from ticks					
			*Rickettsia* sp.	CLB	*Coxiella burnetii*	*Borrelia* sp.	Anaplasmataceae	*Bartonella* sp.
*Ixodes ricinus*	183	MF, n = 169; Switzerland n = 4; UK, n = 2; Italy, Russia, Croatia, the Netherlands, Sweden, Spain, Latvia, or Germany, n = 1	*R.* *helvetica*, n = 2; *R.* *monacensis*, n = 1; *Rickettsia* sp., n = 7	10	0	*B. afzelii*, n = 3; *B. miyamotoi*, n = 2; *Borrelia* sp., n = 11	*Wolbachia* sp., n = 2; *Anaplasma* *phagocytophilum*, n = 1; undetermined, n = 7	0
*I*. *hexagonus*	4	MF, n = 4	0	2	0	0	0	0
*I*. *frontalis*	1	MF, n = 1	0	1	0	0	0	0
*Ixodes* sp.	9	MF, n = 3; UK, n = 2; Switzerland, n = 4	*Rickettsia sp.* 1	3	0	*Borrelia sp.* 1	0	0
*Dermacentor marginatus*	113	MF, n = 113	*R*. *raoultii*, n = 21*; R*. *slovaca*, n = 17; *Rickettsia* sp., n = 11	95	1	0	0	0
*D*. *reticulatus*	5	MF, n = 5	*R*. *raoutlii*, n = 1	1	0	0	0	0
*Dermacentor* sp.	18	MF, n = 18	*R*. *raoultii*, n = 1; *Rickettsia* sp*.*, n = 7	16	1	0	0	0
*Rhipicephalus sanguineus*	52	MF, n = 51; Egypt, n = 1	*R*. *massiliae*, n = 8; *Rickettsia* sp., n = 3	49	1	0	*Ehrlichia canis*, n = 1	0
*R*. *pusillus*	9	MF, n = 9	*R*. *sibirica* *mongolitimoniae*, n = 6; *R.* *massiliae*, n = 3	5	3	0	0	0
*R*. *bursa*	4	MF, n = 4	*R*. *barbariae*, n = 1	3	0	0	0	0
*Rhipicephalus* sp.	2	MF, n = 2	*Rickettsia* sp., n = 1	2	0	0	0	0
*Hyalomma marginatum*	2	MF, n = 2	*Rickettsia* sp., n = 1	0	0	0	0	0
*H*. *aegyptium*	1	Turkey, n = 1	0	0	0	0	0	0
*Hyalomma* sp.	5	MF, n = 2; Greece, n = 3	*R*. *africae*, n = 1	1	0	0	0	0
*Amblyomma variegatum*	3	Guadeloupe, n = 3	*Rickettsia* sp., n = 1, *R*. *africae*, n = 1	2	0	0	0	0
*A*. *hebraeum*	1	South Africa, n = 1	0	0	0	0	0	0
*A*. *mixtum*	1	Cuba, n = 1	*R*. *amblyommatis*, n = 1	1	0	0	0	0
*A*. *oblongoguttatum*	1	Guadeloupe, n = 1	0	0	0	0	0	0
*Argas reflexus*	2	MF, n = 2	0	2	0	0	0	0
*Haemaphysalis concinna*	1	Belgium, n = 1	0	1	0	0	0	0
*H. punctata*	1	MF, n = 1	0	1	0	0	0	0

*Ticks (n = 418) were sent to the Institut Hospitalo-Universitaire Méditerranée Infection in Marseille, France, during 2014–2021 for identification of tick and bacteria species by using mass spectrometry (ticks) and molecular and serologic methods (bacteria). CLB, *Coxiella*-like bacteria; MF, metropolitan France.

### Patients

For each tick received, we collected clinical and epidemiologic data for the patient who was bitten. We collected information on sex, age, date, geographic origin of exposure, symptoms, and antimicrobial drug treatment. The seasonality of tick bites was described only for ticks from metropolitan France, also known as European France, the area of France which is geographically in Europe and includes the Mediterranean island of Corsica. For symptomatic patients, medical consultation and laboratory testing for TBDs was offered to patients who were within a reasonable geographic distance, and advice was given to the patient’s clinician, when they could be reached. 

For laboratory testing, we screened 100 μL of acute-phase serum and, when possible, convalescent serum collected >2 weeks later by using indirect immunofluorescence assays for antigens of spotted fever group *Rickettsia* spp., *Bartonella* *quintana*, *B*. *henselae*, *Borrelia* spp., *F*. *tularensis*, *C*. *burnetii* phase I and II, and *Anaplasma* *phagocytophilum* (*8*). We performed an ELISA for *Borrelia* *burgdorferi* sensu lato and then Western blot if the ELISA was positive (*14*). For acute Q fever (*C*. *burnetii*), we used cutoff titers of 1:200 for phase II IgG and 1:50 for phase II IgM (*15*). For other bacteria, cutoff values were 1:64 for IgG and 1:32 for IgM (*8*). For some patients, we analyzed blood (collected in EDTA tubes, 200 μL), skin biopsy, or eschar swab samples for tickborne pathogens. We extracted DNA from those patient samples and performed quantitative or standard PCR for tickborne pathogens by using the same primers described for ticks (Appendix Table). 

## Results

### Tick Identification

We analyzed a total of 418 ticks removed from 359 patients. The number of tick bites per patient ranged from 1–16. The most frequent tick species identified were *Ixodes* (197 specimens, 47%), *Dermacentor* (136 specimens, 33%), and *Rhipicephalus* (67 specimens, 16%). (Table 1).

We identified 247/254 (92%) ticks by using MALDI-TOF mass spectrometry and 165/179 (92.2%) ticks by using molecular methods at the species level. We did not observe identification discrepancies between MALDI-TOF mass spectrometry and molecular methods. Morphologic identification was performed for 86 (20%) ticks, and we observed congruent species level identification with either MALDI-TOF mass spectrometry or molecular methods, when performed.

### Tick Distribution and Seasonality

**Figure 1:**
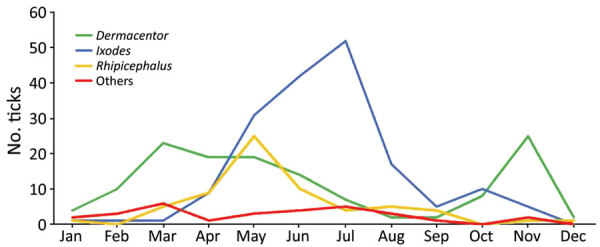
Tick seasonality in study of bacterial agents detected in 418 ticks removed from humans during 2014–2021, France. Overall prevalence of *Dermacentor*, *Ixodes*, *Rhipicephalus*, and other tick species in metropolitan France (n = 387), which includes Corsica, during January–December is indicated. The ticks were among those sent to the Institut Hospitalo-Universitaire Méditerranée Infection in Marseille, France, and identified by using matrix-assisted laser desorption/ionization time-of-flight mass spectrometry or sequencing PCR products.

In metropolitan France, 78% of tick bites occurred in March through August during 2014–2021. Bites from *Ixodes* spp. were more frequent during the summer, bites from *Rhipicephalus* spp. occurred mainly at the end of spring, and bites from *Dermacentor* spp. occurred mainly in spring and autumn (Figure 1).

In metropolitan France, *Ixodes* spp*.* were the most frequently observed ticks, except in southern France, where *Dermacentor* spp. were most frequent **(**Table 1). *Rhipicephalus* spp. ticks originated from southern and eastern France, and 4 *Hyalomma* spp. ticks were received from southern France. Three *Amblyomma* spp. ticks were received from an overseas territory (Guadeloupe) of France, and 28 ticks were received from other countries.

### Bacteria Identified in Ticks

**Figure 2:**
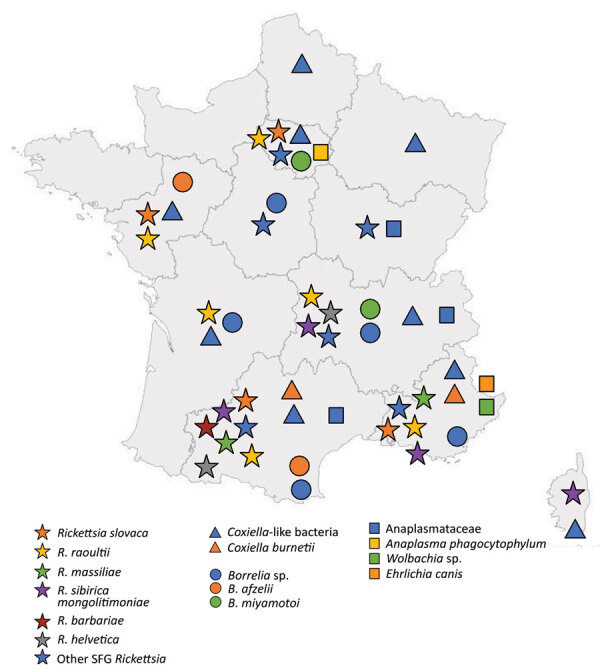
Geographic origin of ticks and identification of tickborne bacteria in study of bacterial agents detected in 418 ticks removed from humans during 2014–2021, France. Symbols indicate tick species and tickborne bacteria identified from locations in metropolitan France, including Corsica. Ticks were sent to the Institut Hospitalo-Universitaire Méditerranée Infection in Marseille, France, and identified by using matrix-assisted laser desorption ionization time-of-flight mass spectrometry. Bacteria carried by the ticks were isolated and identified by PCR or serologic methods at the institute. Of the ticks evaluated, 387 were from metropolitan France; 3 from Guadeloupe, a territory of France in the West Indies; and 28 from other countries.

We detected bacterial DNA in 242/418 (58%) ticks received (Table 1). Co-infections were frequent; 78 ticks were simultaneously infected by 2 bacteria species, and 3 ticks were simultaneously infected by 3 bacteria species. Most co-infections were caused by *Rickettsia* spp. and *Coxiella*-like bacteria (77 ticks). We analyzed the geographic distribution of bacteria found in ticks from metropolitan France (Figure 2).

For ticks from other countries in Europe, 1 tick was infected with *R*. *africae* (Greece), 1 with *Wolbachia* sp. (United Kingdom), 1 with *Borrelia* sp. (Switzerland), and 1 with *Coxiella*-like bacteria (Belgium). In Guadeloupe, an island of France in the West Indies, 1 *Amblyomma* *variegatum* tick was infected with *R. africae*. In Cuba, 1 *Amblyomma* *mixtum* tick was infected with both *R*. *amblyommatis* and *Coxiella*-like bacteria. Ticks received from Asia and Africa were negative for all tested bacteria (Table 1).

### Patient Characteristics

**Figure 3:**
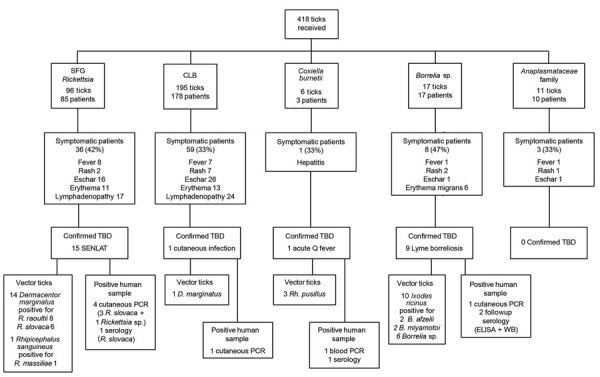
Flow chart of bacteria and tick identification and patient signs/symptoms in study of bacterial agents detected in 418 ticks removed from humans during 2014–2021, France. Ticks were removed from 359 patients and sent to the Institut Hospitalo-Universitaire Méditerranée Infection in Marseille, France, where they were identified by using matrix-assisted laser desorption/ionization time-of-flight mass spectrometry or sequencing PCR products. Bacteria carried by ticks were isolated and identified by PCR or serologic methods at the institute. CLB, *Coxiella*-like bacteria; SENLAT, scalp eschar and neck lymphadenopathy syndrome; SFG, spotted fever group; TBD, tickborne disease; WB, Western blot.

Of the 359 patients who had been bitten by ticks, 137 (38%) were men and 222 (62%) were women. Ages ranged from 1 month to 86 years, and most children (43%, 155) were <10 years of age. We obtained clinical data for 217 (60%) patients; 110 (51%) were asymptomatic and 107 (49%) experienced various symptoms (Figure 3). The most prevalent symptoms were local erythema (37 patients), inoculation eschar (33 patients), lymphadenopathy (27 patients), fever (17 patients), and cutaneous rash (15 patients).

We tested human blood and tissue samples for tickborne pathogenic bacteria (Table 2), and TBD was diagnosed for 26 patients after clinical and biologic investigations (Figure 3). Lyme borreliosis was diagnosed for 9 patients; 6 of those cases had been clinically diagnosed by the original clinician because the patients had typical erythema migrans and were treated with doxycycline. One patient had a skin biopsy that was PCR positive for *Borrelia* sp. and received doxycycline treatment. Lyme borreliosis was diagnosed for the last 2 patients on the basis of seroconversion between acute and follow-up serum samples. We observed cephalic or cervical inoculation eschar and cervical lymphadenopathy corresponding to scalp eschar and neck lymphadenopathy syndrome (SENLAT) after tick bites in 15 patients. Acute hepatitis developed in 1 patient who had positive serologic results (1:400 phase II IgG, 1:50 phase II IgM) and positive blood PCR results for *C*. *burnetii*; acute Q fever was diagnosed, and the patient was treated with doxycycline. One patient had a skin biopsy that was PCR-positive for *Coxiella*-like bacteria and was treated with doxycycline. All patients with a TBD diagnosis had been bitten by ticks that carried pathogenic bacteria. The remaining symptomatic patients did not meet clinical or biologic criteria for a TBD diagnosis.

**Table 2:** Bacterial species identified in different patient samples in study of bacterial agents detected in 418 ticks removed from humans during 2014–2021, France*

Patient sample	Total	Identified bacteria	Bacteria found in tick	Negative samples
Blood†	31	*Coxiella* *burnetii*, n = 1	Yes	30
Cutaneous‡	39	*Rickettsia* *slovaca*, n = 3; *Borrelia* sp., n = 1; *Rickettsia* sp., n = 1; *Coxiella*-like bacteria, n = 1	Yes	30
Serum, acute	47	*Borrelia* sp., n = 3	No	44
Serum, followup	30	*Borrelia* sp., n = 2; *Coxiella* *burnetii*, n = 1; *R*. *slovaca*, n = 1	Yes	26

*Different types of patient samples for which at least 1 tick was also identified were tested for bacteria by using serologic and molecular methods at the Institut Hospitalo-Universitaire Méditerranée Infection, Marseille, France. †Collected in tubes with EDTA. ‡Samples from 33 biopsies and 6 cutaneous swab specimens.

We collected data on antimicrobial drug treatment received by 80 patients, 12 of whom were treated at the IHU-MI. In addition to the patient treated for Q fever, patients at the IHU-MI were treated with doxycycline after being bitten by *Dermacentor* ticks positive for *R*. *raoultii* (2 patients) or *R*. *slovaca* (2 patients) that caused SENLAT. For the 68 patients treated outside the IHU-MI, when a TBD diagnosis was suspected, the managing clinician sometimes began probabilistic antimicrobial drug treatment, which could be continued or suspended according to tick analysis results. The most frequently used antimicrobial drugs after tick bites were doxycycline (26 patients), azithromycin (21 patients), and amoxicillin (18 patients). Other antimicrobial drugs used were pristinamycin, vancomycin, amoxicillin/clavulanic acid, and topical fucidic acid ointment.

## Discussion

We identified 418 ticks that were removed from 359 patients in France by using various methods and emerging tools, such as MALDI-TOF mass spectrometry. Rapid identification of ticks has major clinical implications because different tick species carry different pathogens. Arthropods have historically been identified morphologically and, more recently, by using molecular methods (*16*). Morphologic identification of ticks during routine clinical practice is limited by the availability of appropriate documentation, trained entomologists, and might also be impeded if the arthropod specimen’s preservation state is poor (*17*). MALDI-TOF mass spectrometry has been used for identification of microorganisms since ≈2003. MALDI-TOF mass spectrometry has also been shown to be a robust, reproducible, and time-effective method for identifying arthropod vectors, notably ticks (*9**,**12*); advantages are low running costs and time efficiency compared with molecular methods. Moreover, no specific expertise is required, in contrast to morphologic approaches. In our study, we identified ticks by MALDI-TOF mass spectrometry and successfully applied results to routine diagnoses. Limitations of this method are the need to obtain good quality spectra and availability of an extensive database for reliable identification (*18*). Biomolecular methods also identified tick species efficiently, but those methods can be affected by PCR inhibitors in the ticks (*19*). The combination of MALDI-TOF mass spectrometry, molecular, and morphologic identification methods enabled the complete identification of 92% of ticks in our study. The remaining 8% were only identified at the genus level, often because of insufficient tick material (which limited the number of analyses that could be performed) and limitations of the various diagnostic methods.

Data on tick engorgement status or attachment duration were not available and, thus, not analyzed in our study. Acquisition of those data is needed because engorgement indicates an efficient blood meal, which is more likely to result in bacterial transmission (*20*). Transmission of microorganisms is linked to attachment duration. During the first hours, the tick mainly injects the cement that will enable firm attachment to the host’s skin; transmission of bacterial agents usually occurs 20–24 hours after attachment (*21*).

*Ixodes* spp. ticks are present in every region of France. *I*. *ricinus,* the known vector of Lyme disease in Europe, mostly lives in temperate humid regions and forested areas but can also be found in specific biotopes within the Mediterranean area (*22*). *Ixodes* spp. tick bites can occur throughout the year but have a higher prevalence in summer when tick populations, especially biting nymphs, are at their peak, which is also associated with the highest occurrence of Lyme disease (*23*). We observed that *I*. *ricinus* ticks were frequently infected by *Borrelia* sp. (9%). Nine patients had documented Lyme borreliosis after bites from *I*. *ricinus* ticks infected with *Borrelia* sp., mostly acute infections confirmed either clinically (6 patients with erythema migrans) or by PCR (1 cutaneous specimen) or serology (2 positive follow-up serum samples). The seroconversion period for Lyme borreliosis is 2–4 weeks, and, in early Lyme disease, the diagnosis can be made by the presence of erythema migrans alone without positive serology; serology is frequently negative in the early stage of Lyme disease (*24*). Identifying *Borrelia* sp. DNA in the tick can guide the patient’s treatment and surveillance before seroconversion. Furthermore, 8% of *Ixodes* spp. ticks were infected with spotted fever group *Rickettsia*, including *R*. *helvetica* and *R*. *monacensis,* both emerging pathogens associated with those ticks (*25*). We found *Ixodes* spp. ticks (2 ticks) carried *Wolbachia* sp. bacteria, endosymbionts of many arthropods including ticks (*26*) and not known to be human pathogens. In 1 *I*. *ricinus* tick, we found *A*. *phagocytophilum* bacteria, the cause of human granulocytic anaplasmosis, which can induce fever, cytopenia, and elevated levels of transaminases in the blood. Granulocytic anaplasmosis is diagnosed by using PCR, blood smears, or retrospectively by serology (*27*).

*Dermacentor* spp. ticks are found in various habitats and have high tolerance to temperature variations. In Europe, *D*. *marginatus*, the ornate sheep tick, is most frequently found in Mediterranean areas. *D*. *reticulatus,* the ornate dog tick, is most frequently found in colder northern areas that have high humidity and mild winters. We observed *Dermacentor* tick bite peaks in early spring and autumn and a decrease in summer activity, which is frequently described in temperate Europe (*28*). *Dermacentor* ticks are potential vectors for various human pathogens (*29*) and were associated with SENLAT in 14 cases after bites from *D*. *marginatus* ticks infected with either *R*. *raoultii* or *R*. *slovaca*. *R*. *slovaca* infection was first referred to as TIBOLA (tick-borne lymphadenopathy) (*30*); lymphadenopathy is the most frequent symptom. After a role for *Dermacentor* ticks was found, the name DEBONEL (*Dermacentor-*borne necrosis erythema lymphadenopathy) was proposed (*31*). *R*. *raoultii* was identified as another frequent etiologic agent of lymphadenopathy (*32*), which is caused by local control of infection within the lymph node but is not pathogen-specific. Lymphadenopathy can be caused by other tickborne bacteria, including *C*. *burnetii*, *B*. *burgdorferi*, *B*. *henselae*, and *F*. *tularensis* (*33*). The acronym SENLAT was proposed to provide an accurate clinical description of lymphadenopathy after tick bite (*34*), and diagnosis is based on typical symptoms. In our study, SENLAT occurred in 1 patient after a bite from a *Rh*. *sanguineus* s.l. tick infected with *R*. *massiliae.* SENLAT etiology is determined by detecting bacterial DNA in vector ticks or from eschar swab samples (*35*) because serology sensitivity might be low (12% for *R. slovaca*) and seroconversion might occur only during late stages of infection (*36*).

We also detected a high (83%) prevalence of *Coxiella*-like bacteria in *Dermacentor* ticks. One patient, who was bitten by a *D*. *marginatus* tick and had a cutaneous rash and an eschar, was PCR-positive for *Coxiella*-like bacteria in a cutaneous specimen; the bacteria were also found in the tick. However, pathogenic potential of *Coxiella*-like bacteria in humans remains unclear.

*Rh,* *sanguineus* s.l. ticks (brown dog ticks) are found in proximity to dogs, which are their primary feeding hosts (*37*). In our study, we identified *Rh*. *sanguineus* s.l. ticks mostly in southern France, where they are endemic. *Rhipicephalus* ticks were more frequently found at the end of spring, although previous studies have described peak activity during the summer months, potentially because of warmer temperatures during spring months in recent years. Although *Rhipicephalus* ticks are active during May–November, human bites are reported more frequently during the warmer months, most likely because warmer weather increases their propensity to bite other hosts, including humans (*38*). *Rh*. *sanguineus* s.l. ticks are vectors for *R*. *conorii* *conorii,* the agent causing Mediterranean spotted fever (*39*). None of the 418 ticks in our study was positive for *R*. *conorii* *conorii*, in line with a study from Spain, where 2,229 *Rh*. *sanguineus* ticks were negative for that bacteria (*40*). *R*. *conorii* *conorii* infections of *Rh*. *sanguineus* s.l. ticks might vary in the wild from 1 specific setting to another and have a small focus, low propensity for diffusion, and potentially understudied vertebrate reservoir and environmental requirements (*39*).

Several species of *Rhipicephalus* ticks can carry *R. massiliae* (*41*). In our study, we found *R*. *massiliae* in 8 *Rh*. *sanguineus* s.l. and 3 *Rh*. *pusillus* ticks from southern France. Since the first case reported in Italy in 2005, only a few human cases of *R. massiliae* infection have been reported, which causes symptoms similar to those of Mediterranean spotted fever or SENLAT (*25*). We found *R. sibirica mongolitimonae*, which was associated with lymphangitis-associated rickettsiosis (*42*), in 5 *Rh. pusillus* ticks, occurring more frequently in the spring and summer in France (*25*). We found *Candidatus* Rickettsia barbariae in 1 *Rhipicephalus* sp. tick, which has been detected previously in ticks in France and elsewhere, but its pathogenicity is unknown (*13*). *Rhipicephalus* ticks were also frequent (88%) carriers of *Coxiella*-like bacteria. Those endosymbionts are part of the microbiome of *Rhipicephalus* and other ticks and might promote tick development and fertility (*43*).

We report 1 patient who had acute Q fever that was documented by seroconversion and PCR of a blood sample and complicated by hepatitis; the patient was bitten by 3 *Rh. pusillus* ticks, all of which were infected with *C. burnetii*. Ticks are competent vectors for *C. burnetii* in experimental models, but only a few cases of Q fever caused by tick bites have been reported. The main route of human infection is through exposure to infected ruminants and their products via aerosols or direct contact (*44*). The clinical manifestations of Q fever can vary from influenza-like symptoms in acute disease to persistent focalized infections, such as endocarditis and vascular infections (*16*).

*Hyalomma* ticks can be found in Asia, Africa, and Europe and are of medical and veterinary significance in tropical regions (*45*). In our study, 1 patient was bitten in Greece by a *Hyalomma* sp. tick that was positive for *R*. *africae*, the etiologic agent of African tick bite fever, known to be endemic in sub-Saharan Africa and the West Indies. *Hyalomma* ticks have been reported to carry *R*. *africae* (*46*), but no proof exists for their vectorial competence for African tick bite fever. Indeed, the main recognized vectors of the disease are *Amblyomma* spp. ticks, such as *A. variegatum* (*25*,*47*). We found that 1 *A. variegatum* tick from a patient in Guadeloupe carried *R. africae*. All tick bites by *Amblyomma* spp. reported in our study occurred in tropical territories, but no cases of related diseases were diagnosed. Of note, we detected *R*. *amblyommatis* in an *A*. *mixtum* tick from Cuba; this spotted fever group rickettsia is known to infect *Amblyomma* ticks, but its pathogenicity in humans is unknown (*25*).

In conclusion, our study underscores the large number of tick species that can bite humans and bacteria species that ticks can carry, including recognized and unknown pathogens. Furthermore, MALDI-TOF mass spectrometry is an efficient technique for identifying ticks in diagnostic settings and has recently been evaluated in terms of its ability to detect the infectious status of ticks (*48*). Our study enabled the reorganization of our laboratory for optimal specimen analysis. Currently, ticks are photographed first by laboratory technicians in accordance with specific guidelines if an entomologist is unavailable. Ticks are then identified by using MALDI-TOF mass spectrometry. If the quality of the mass spectrometry spectrum is low or identification is doubtful, the tick is identified by using PCR and then sequencing. Detection of bacteria by PCR is conducted simultaneously. Most ticks are known vectors of various TBDs, and identification of the tick species and bacteria they carry is a first step in disease diagnosis for the patient who has been bitten. Of note, transmission of a bacterial agent through the bite of an infected tick does not occur systematically, because transmission of bacteria is dependent on the duration of tick attachment. In nonexpert settings, such as local laboratories, rapid identification of ticks and the pathogens they carry can lead to expedited decisions to treat patients if the tick is infected. In addition, knowledge of local tick and bacteria ecology might influence patient care strategies. Knowing that a specific TBD is prevalent in a region where tick bites occurs necessitates close surveillance of the patient for disease symptoms, and, in TBD hyperendemic areas, patients might benefit from preventive antimicrobial drug treatment after a tick bite (*49*). Rapid detection and identification of ticks and tickborne bacteria by using a combination of MALDI-TOF mass spectrometry, molecular methods, and serology can substantially contribute to early TBD diagnoses and treatment.

Appendix Additional information for bacterial agents detected in 418 ticks removed from humans during 2014–2021, France.
