# Supplementary material for: Bacterial Agents Detected in 418 Ticks Removed from Humans during 2014–2021, France
Source: Emerg Infect Dis. 2023 Apr;29(4):701–10. doi: 10.3201/eid2904.221572 (PMC10045685; doi:10.3201/eid2904.221572)
Supplement: Appendix — Additional information for bacterial agents detected in 418 ticks removed from humans during 2014–2021, France. [file 22-1572-Techapp-s1.pdf]

Article DOI: https://doi.org/10.3201/eid2904.221572

EID cannot ensure accessibility for supplementary materials supplied by authors. Readers who have difficulty accessing supplementary content should contact the authors for assistance.

Bacterial Agents Detected in 418 Ticks Removed from Humans during 2014–2021, France

Appendix

References

1. Dahmani M, Davoust B, Benterki MS, Fenollar F, Raoult D, Mediannikov O. Development of a new PCR-based assay to detect Anaplasmataceae and the first report of *Anaplasma phagocytophilum* and *Anaplasma platys* in cattle from Algeria. Comp Immunol Microbiol Infect Dis. 2015;39:39–45. PubMed https://doi.org/10.1016/j.cimid.2015.02.002

2. Mediannikov O, Fenollar F. Looking in ticks for human bacterial pathogens. Microb Pathog. 2014;77:142–8. PubMed https://doi.org/10.1016/j.micpath.2014.09.008

3. Parola P, Diatta G, Socolovschi C, Mediannikov O, Tall A, Bassene H, et al. Tick-borne relapsing fever borreliosis, rural Senegal. Emerg Infect Dis. 2011;17:883–5. PubMed https://doi.org/10.3201/eid1705.100573

4. Willems H, Thiele D, Frölich-Ritter R, Krauss H. Detection of *Coxiella burnetii* in cow's milk using the polymerase chain reaction (PCR). Zentralbl Veterinarmed B. 1994;41:580–7. PubMed https://doi.org/10.1111/j.1439-0450.1994.tb00267.x

5. Angelakis E, Roux V, Raoult D, Rolain J-M. Real-time PCR strategy and detection of bacterial agents of lymphadenitis. Eur J Clin Microbiol Infect Dis. 2009;28:1363–8. PubMed https://doi.org/10.1007/s10096-009-0793-6

6. Socolovschi C, Kernif T, Raoult D, Parola P. *Borrelia*, *Rickettsia*, and *Ehrlichia* species in bat ticks, France, 2010. Emerg Infect Dis. 2012;18:1966–75. PubMed https://doi.org/10.3201/eid1812.111237

Appendix Table. Sequences of the PCR primers and probes used for bacterial detection*

Bacteria	Gene	Primer	Sequence (5′–3′)	Reference
Anaplasmatacae	*23S*	F	TGACAGCGTACCTTTTGCAT	(*1*)
		R	GTAACAGGTTCGGTCCTCCA	
		P	6FAM-GGATTAGACCCGAAACCAAG	
Bartonella spp.	*ITS*	F	GGGGCCGTAGCTCAGCTG	(*2*)
		R	TGAATATATCTTCTCTTCACAATTTC	
		P	6FAM-CGATCCCGTCCGGCTCCACCA	
Borrelia spp.	*16S*	F	AGCCTTTAAAGCTTCGCTTGTAG	(*3*)
		R	GCCTCCCGTAGGAGTCTGG	
		P	6FAM-CCGGCCTGAGAGGGTGAACGG	
Coxiella burnetii	*IS111*	F	CAAGAAACGTATCGCTGTGGC	(*4*)
		R	CACAGAGCCACCGTATGAATC	
		P	6FAM-CCGAGTTCGAAACAATGAGGGCTG	
Coxiella-like	*16S*	F	ACCTACCCTTGACATCCTCGGAA	(*2*)
		R	GCAACTAAGGACGAGGGTTG	
		P	6FAM-CAGCTCGTGTCGTGAGATGT	
Francisella tularensis	*yqaB*	F	GCTGATGATAATCACCCGAGTAAAA	(*5*)
		R	TCCTGGAACACCATCTTCAAAAA	
		P	6FAM-CCCCAAGGCGTTTACTTTGATCGCA	
Rickettsia spp.	*gltA*	F	GTGAATGAAAGATTACACTATTTAT	(*6*)
		R	GTATCTTAGCAATCATTCTAATAGC	
		P	6FAM-CTATTATGCTTGCGGCTGTCGGTTC	

*6FAM, 6-carboxyfluorescein; F, forward; P, probe; R, reverse.
